# Induction of Krüppel‐like factor 2 reduces K/BxN serum‐induced arthritis

**DOI:** 10.1111/jcmm.14041

**Published:** 2018-12-03

**Authors:** Manjusri Das, Dipranjan Laha, Suman Kanji, Matthew Joseph, Reeva Aggarwal, Obiajulu H. Iwenofu, Vincent J. Pompili, Mukesh K. Jain, Hiranmoy Das

**Affiliations:** ^1^ Department of Internal Medicine The Ohio State University Medical Center Columbus Ohio; ^2^ Department of Pharmaceutical Sciences School of Pharmacy Texas Tech University Health Sciences Center Amarillo Texas; ^3^ Department of Pathology College of Medicine The Ohio State University Columbus Ohio; ^4^ Department of Internal Medicine Case Western Reserve University Cleveland Ohio

**Keywords:** HDAC inhibitors, inflammation, KLF2, monocytes, osteoclasts, rheumatoid arthritis

## Abstract

Krüppel‐like factor 2 (KLF2) critically regulates activation and function of monocyte, which plays important pathogenic role in progressive joint destruction in rheumatoid arthritis (RA). It is yet to be established the molecular basis of KLF2‐mediated regulation of monocytes in RA pathogenesis. Herein, we show that a class of compound, HDAC inhibitors (HDACi) induced KLF2 expression in monocytes both *in vitro* and *in vivo*. KLF2 level was also elevated in tissues, such as bone marrow, spleen and thymus in mice after infusion of HDACi. Importantly, HDACi significantly reduced osteoclastic differentiation of monocytes with the up‐regulation of KLF2 and concomitant down‐regulation of matrixmetalloproteinases both in the expression level as well as in the protein level. In addition, HDACi reduced K/BxN serum‐induced arthritic inflammation and joint destruction in mice in a dose‐dependent manner. Finally, co‐immunoprecipitation and overexpression studies confirmed that KLF2 directly interacts with HDAC4 molecule in cells. These findings provide mechanistic evidence of KLF2‐mediated regulation of K/BxN serum‐induced arthritic inflammation.

## 1 INTRODUCTION

Rheumatoid arthritis (RA) is a chronic autoimmune disorder manifested by progressive destructions of synovial joints, articular cartilages and bones.^1^ It is the most common form of inflammatory arthritis resulting in disability.^2^ Even though many current drugs have the potential to reduce pain at the early stage of this disease, the long‐term effect are highly associated with undesired side effects.^3^ Immune cells are critical in initiating pathogenesis of RA.^4^ The recruitment of monocytes to the inflammatory sites triggers the secretion of proinflammatory effectors leads to tissue destruction.^5^ Recruited monocytes differentiate into synovial tissue macrophages and produce a number of inflammatory mediators that activate surrounding resident cells.^6^ They also differentiate into osteoclasts, which are mainly responsible for subchondrial bone destruction in RA.^7^ These osteoclasts can be formed not only from the mature tissue macrophages but also from the immature cells of the monocytes‐macrophage lineage. Therefore, monocytes are central to the pathophysiology of RA, and they have been found to be activated in RA patients too.^8^ Hence, identifying a better regulatory mechanism for monocyte activation in RA will help in creating better management of this disease using more specific targets.

Krüppel‐like transcription factor (KLF)s are involved in regulation of terminal differentiation of several immune cells. Lung KLF (LKLF) or KLF2 plays a significant regulatory role in haematopoietic cell biology including cell quiescence, cell proliferation, differentiation and survival.^9^, ^10^ KLF2 also regulates myeloid cell activation and function.^11^ During inflammation, KLF2 acts as a potent inhibitor of transcription factors like NF‐κB and AP1, as well as of hypoxia‐related HIF‐1α protein.^12^ Importantly, KLF2 is essential for embryonic erythropoiesis and KLF2‐deficient mice are embryonic lethal because of the leaky blood vessels and haemorrhages.^13^

Krüppel‐like factor 2 is a negative regulator of monocyte activation and osteoclast differentiation, directs them towards quiescent state.^11^, ^14^ The current study focuses on assessing role of a special class of pharmacological compounds, histone deacetylase inhibitors (HDACi) in modulation of KLF2 in monocytes both in vitro and in vivo. HDACi have been used clinically for the treatment of various cancer malignancies.^15^ HDACs are well known for their role in chromatin remodelling,^16^ and also regulate large number of non‐histone proteins, which controls cell processes associated to various disease conditions.^17^ Evidence show that HDACi controls NFκB‐driven inflammatory responses through cell cycle arrest.^18^ However, the effect of HDACi on inflammatory response varies according to the cell type and stimulus. As cell cycle arrest leads to cell quiescence, and KLF2 is induced upon cell quiescence, we were interested to know whether HDACi can mediate induction of KLF2. Among the common HDACi are suberoylanilide hydroxamic acid (SAHA), entinostat (SNDx‐275 or MS‐275) and Trichostatin A (TSA) are of consideration. In addition, it is yet to be defined how HDACi regulate myeloid KLF2 and RA pathogenesis. To investigate the efficacy of HDACi against RA progression and to delineate the mechanistic evidence have driven us to explore this intrigued pathogenesis.

## 2 MATERIALS AND METHODS

### 2.1 Isolation and culture of monocytes from murine bone marrow

Bone marrow (BM) cells were isolated from the femurs of 6‐ to 8‐week‐old C57BL/6 mice, and cultured in DMEM medium (Sigma) supplemented with 10% foetal bovine serum (ThermoFisher, Grand Island, NY, USA) at 37°C in a 5% CO_2_ atmosphere for plastic adherence. Adhered monocytes were either stimulated with suberoylanilide hydroxamic acid (SAHA from Sigma), entinostat (SNDx‐275 or MS‐275; Cayman Chemical Company, Ann Arbor, MI, USA) and Trichostatin A (TSA from Sigma) for either 24 or 48 hours or cultured without stimulus (DMSO, vehicle) for control.

### 2.2 *In vivo* stimulation of mice with HDACi

To investigate whether systemic stimulation with HDACi to the mice have any effect on immune cell‐rich tissues, three mice per group were injected intra peritoneal (i.p.) with MS275 (5 mg/kg BW in 50 μL DMSO) and TSA (10 mg/kg BW in 50 μL DMSO) or DMSO (50 μL, as a control). After 24 hours of HDACi injections, BM, spleen and thymus were harvested. A part of the tissues was homogenized and RNA was extracted, and another part was subjected to protein extraction.

### 2.3 Isolation of human monocytes

Fresh human peripheral blood (n = 6) was collected with an approved IRB and written consent from donors from The Ohio State University Medical Center, Columbus, OH and processed following earlier described protocol.^11^, ^19^ In, brief, peripheral blood mononuclear cells were isolated from freshly collected blood using Ficoll‐Paque density centrifugation. CD14^+^ cells were isolated by using an AutoMACS device, CD14^+^ antibody and reagents (all from Miltenyi Biotec, San Diego, CA, USA) following earlier established protocol.^11^, ^20^ A part of the CD14^+^ cells was used for RNA extraction, and another part was subjected to protein extraction.

### 2.4 Real time RT‐PCR analysis

Total RNA was isolated from BM‐derived monocytes, RAW 264.7 cell (ATCC, Manassas, VA, USA) and human peripheral blood‐derived monocytes after 24 hours of HDACi stimulation, or murine tissues after infusion with HDACi to mice using a RNeasy Kit (Qiagen, ThermoFisher). One microgram of RNA was used for synthesis of cDNA using oligo dT (Invitrogen, ThermoFisher) primer. Real‐time RT‐PCR was performed with 1 μL of cDNA for the gene‐specific primers of KLF2, matrixmetalloproteinase 3 (MMP3), MMP9 and MMP13, keeping GAPDH as an internal control using a standard SYBR green Taqman protocol, and a real‐time PCR machine (MX3000P; Stratagene, Santa Clara, CA, USA). Relative fold‐expression levels of stated genes were measured considering respective unstimulated cells or tissues (added DMSO) as controls. Experiments were performed in triplicate and were repeated at least three times.

### 2.5 Osteoclast differentiation

Bone marrow cells were collected from femurs of mice were induced for osteoclastic differentiation in vitro in the presence or absence of HDACi (SAHA or MS‐275 both 10 nmol/L). In, brief, BM cells were cultured overnight at 37°C incubator with 5% CO_2_ in αMEM containing 10% heat inactivated foetal bovine serum in the presence of 20 ng/mL M‐CSF (R & D Systems, Minneapolis, MN, USA). Next day, non‐adherent cells were collected and incubated for an additional 6 days in αMEM medium with 20 ng/mL M‐CSF, and 50 ng/mL GST‐RANKL.^21^ Fresh medium was replaced every alternate day and HDACi was added to the medium as needed. At days 3 and 6 of differentiation, the cells were stained for TRAP staining using an acid phosphatase, leukocyte; Trap Staining Kit (Sigma) and was viewed and imaged with a fluorescence microscope (Nikon, Axioplan2; Carl Zeiss). TRAP‐positive multinucleated cells (3 nuclei, DAPI positive) were counted as osteoclast‐like cells.

### 2.6 Induction of arthritis

K/BxN mice were generated by crossing KRN, TCR‐transgenic B6 mice (kind gift from Dr. Diane Mathis, Harvard Medical School, Boston, MA, USA) with NOD mice (Jackson Laboratory, Bar Harbor, ME, USA) following established protocol.^22^ K/BxN serum was collected from 6‐ to 8‐week‐old arthritic K/BxN mice and pooled for each experiment. Four groups (n = 8 for each group) of mice (C57BL/6 background 6‐8 weeks old, both male and female) were induced by i.p. injection of 150 μL of K/BxN serum on days 0 and 2 following earlier established protocol.^23^ Three groups were treated with HDAC inhibitors during the course of arthritis development for 7 days (control = no treatment; treatment with SAHA, 10 mg/kg body weight; treatment with SAHA, 30 mg/kg body weight; and treatment with MS275, 5 mg/kg body weight). Mice were observed everyday and measured for ankle thickness using a microcaliper (Mitutoyo, MSC Industrial Supply Co. USA), and finally, sacrificed on day 8 following first injection of K/BxN serum administrations. Hindlimbs were harvested and were subjected to histopathological analysis.

### 2.7 Histological assessment of arthritis

Arthritis was assessed by histological examination as described,^14^ with some modifications. Limbs were fixed in periodate‐lysine‐paraformaldehyde for overnight and decalcified in 10% EDTA (BDH Chemicals, Victoria, Australia) and 7.5% polyvinylpyrolidone (Sigma) in Tris buffer (pH 6.95) for 7‐10 days and processed for paraffin embedding. Tissues were sectioned with 5 μmol/L thickness, placed on aminoalkylsilane‐coated slides, and stained for routine histology with haematoxylin and eosin (H&E). Five defined pathological features were graded for severity from 0 (normal) to 5 (severe), according to established protocol,^24^ and in a blinded manner. Soft tissue inflammation, assessed in the infrapatellar fat pads, joint capsule, and the area adjacent to the periosteal sheath, was graded according to the extent of cellular infiltration and angiogenesis. Joint space exudate was identified as leucocytes scattered discretely or in aggregates in the joint space. Synovitis (synovial hyperplasia) was defined as hyperplasia of the synovium, but did not include pannus formation. Pannus was defined as hypertrophic synovial tissue forming a tight junction with the articular surface. Evaluation of cartilage and bone damage was based on loss of cartilage matrix, disruption and loss of cartilage surface, and the extent and depth of the subchondral bone erosion.^25^ A trained pathologist, Department of Pathology, The Ohio State University Medical Center performed the histomorphometric analyses from multiple H&E sections.

### 2.8 Western blotting

Total protein analysis was performed with the standard Western blot (WB) method with equal amount of proteins isolated from human primary monocytes or RAW 264.7 cells that were stimulated for 24 hours with HDACi or from homogenized murine tissues after infusion with HDACi to the mice for 24 hours were fractionated in a SDS‐polyacrylamide gel. WB analysis was performed with KLF2 (Abcam Cambridge, MA, USA and Santa Cruz Biotech, Dallas, TX, USA), MMP9, AcHistone3, HDAC1, HDAC3, HDAC4, p65, β‐actin and GAPDH (all from Cell Signaling Technology, Danvers, MA, USA) antibodies.

### 2.9 Co‐immunoprecipitation assays

COS‐7 (ATCC) cells were transfected with the indicated expression plasmids (KLF2‐Myc and HADC1‐4 all Flag tagged) using Lipofectamine 2000 (ThermoFisher Scientific) transfection reagent following the manufacturer's protocol, and harvested in RIPA buffer after 48 hours of transfection. One milligram of each lysates was subjected to immunoprecipitation with 5 μg of αMyc monoclonal antibody (ThermoFisher) at 4°C for 2 hours followed by incubation with protein A/G Sepharose beads (Abcam Inc) for overnight at 4°C. The beads were washed and proteins were separated by SDS‐PAGE as previously described^11^ and developed with anti‐Flag antibody (Sigma).

### 2.10 KLF2 overexpression in RAW264.7 cells by adenoviral infection

RAW 264.7 cells were infected with control (Ad‐GFP) or KLF2 (Ad‐KLF2) virus at 50 MOI. In general, ~40%‐50% infection was achieved within 24‐48 hours of incubation with adenovirus at which time cells were harvested and lysed for protein analysis.

### 2.11 Statistical analysis

Values were expressed as mean ± SEM and statistical analysis was performed by ANOVA. Student's *t* test was performed and the results were considered significant when *P*‐values were <0.05.

## 3 RESULTS

### 3.1 Effect of HDAC inhibitors on KLF2 expression in monocytes

**Figure 1:**
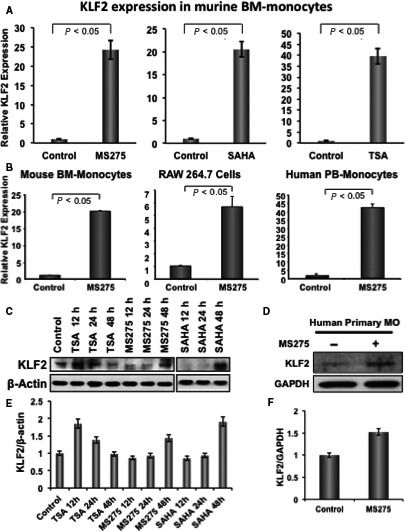
HDAC inhibitor induces KLF2 expression in monocytes. (A) Real‐time RT‐PCR was performed to evaluate the level of relative KLF2 expression in RNA isolated from mouse primary bone marrow‐derived monocytes (n = 3), after treating with 10 nmol/L of various HDACi (MS275, SAHA or TSA) for 24 h, and without stimulus (DMSO) was considered as a base line control of KLF2 expression, and the relative fold expression was graphically presented. GAPDH was considered as an internal control. Experiments were performed in triplicate. (B) Similar experiment was performed with mouse primary bone marrow‐derived monocytes (n = 3), mouse monocyte cell line (RAW 264.7) and human primary peripheral blood‐derived monocytes (n = 3) after treating with 10 nmol/L HDACi (MS275) for 24 h, and data are presented graphically. (C, D) Similar experiments were performed with mouse bone marrow monocytes, RAW 264.7, and human primary peripheral blood‐derived monocytes after stimulation with 10 nmol/L of TSA, or MS275 or SAHA, and proteins were evaluated at various time‐points as stated, and evaluated for KLF2 expression using Western blot methods using β‐actin as an internal control. (E, F) Quantified Western blot data of figures C, D are presented graphically

To test the effect of HDACi on KLF2 gene expression, three different types of HDACi (MS275, SAHA and TSA) at various concentrations (1‐50 nmol/L) were added to the murine monocytic cells (RAW 264.7), and DMSO was added as a vehicle control. After 24 hours of incubation, RNA was isolated from harvested cells, were subjected to KLF2 gene expression using the quantitative RT‐PCR method, and simultaneously cell viability was verified by a Trypan blue exclusion test. All HDACi induced KLF2 at various degrees (2‐ to 17‐fold, Figure S1); however, 50 nmol/L concentrations reduced cell viability significantly (data not shown). For subsequent studies sublethal dose, 10 nmol/L of HDACi were used for testing in mouse primary BM monocytes. Similar to the cell line, all HDAC inhibitors also induced KLF2 in primary BM monocytes at various degrees from 20‐ to 40‐fold (Figure 1A). We next tested one of the HDACi (MS275) in mouse BM monocytes or RAW 264.7 or human peripheral blood‐derived (PB‐) monocytes. Cells were incubated with 10 nmol/L HDACi (MS275) and DMSO was considered as basal control similar to the earlier experiments. Isolated RNA was subjected to real‐time RT‐PCR for KLF2 expression, and the relative fold expression was graphically presented considering GAPDH as an internal control (Figure 1B). A 20‐fold higher expression of KLF2 in mouse BM monocytes were found with MS275 compared to the untreated (control) cells. Whereas the KLF2 expression level was almost sixfold higher in RAW 264.7 cell after incubation with MS275 compared to the control cells. Interestingly, in human PB‐monocyte, it showed a 43‐fold elevation in KLF2 expression after treatment with MS275 compared to untreated cells. To confirm, KLF2 expression was translated into protein, WB analysis was performed. WB data revealed that the protein levels of KLF2 were markedly higher in both human primary PB‐monocytes (Figure 1C) and murine transformed monocytic cells after stimulation with various HDACi at multiple time‐points compared to unstimulated control cells (Figure 1C). The expression level of KLF2 was not consistent with all HDACi in the context of time‐points. For example, highest expression was noticed with TSA at 12 hours, whereas with SAHA and MS275, it was at 48 hours time‐point. The Western blot data were quantified and shown graphically (Figure 1E,F). Nevertheless, these data indicated that HDACi induced KLF2 expression in monocytes both in the transcriptional level as well in the protein level.

### 3.2 Tissue expressions of KLF2 in mice after injection with HDAC inhibitor

**Figure 2:**
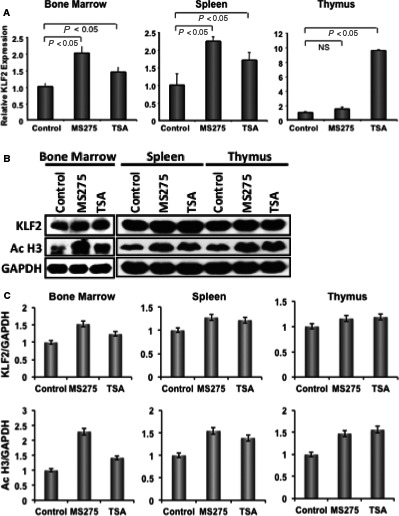
HDAC inhibitor induces KLF2 expression in various tissues in mice. (A) Three mice per group were injected (ip) with HDACi, and after 24 h bone marrow, spleen and thymus were harvested. Tissues were homogenized and part was subjected to real‐time RT‐PCR to evaluate the level of relative KLF2 expression in RNA. Vehicle control injected mice was considered as a base line control for KLF2 expression and the relative fold expression was graphically presented for various tissues. GAPDH was considered as an internal control. PCR experiments were performed in triplicate. (B) Other part of the tissues was subjected to protein extraction, and followed by Western blot analysis for KLF2 expression. The levels of KLF2 expression and acetylated Histone 3 were shown along with GAPDH (as an internal control). (C) Quantified Western blot data of stated molecules are presented graphically

To investigate whether HDACi injection to the mice has any effect on KLF2 level in immune rich tissues, mice were injected (i.p.) with MS275 (5 mg/kg BW in 200 μL DMSO) and Trichostatin A (TSA, 10 mg/kg BW in 200 μL DMSO) or DMSO (as a control). After 24 hours of HDACi injections, BM, spleen and thymus tissues were harvested and analysed. Real‐time RT‐PCR analysis revealed that significant higher level of KLF2 expression in all tissues after injection with both HDAC inhibitors (MS275 or TSA) compared to vehicle injected controls, except MS275 in thymus, where little elevation of KLF2 was noticed (Figure 2A). Western blot analysis (Figure 2B) for KLF2 revealed that the level of KLF2 was markedly higher in all tissues where animals were injected with either of the HDAC inhibitors (MS275 or TSA) compared to the control animals. We also investigated whether HDAC inhibitors activate histones for transcriptional activities. Indeed, we found that level of acetylated Histone 3 (Ac‐H3) was markedly elevated in all tissues of animals that were injected with either of the HDAC inhibitors (MS275 or TSA) compared to the control animals. The Western blot data were quantified and shown graphically (Figure 2C). These data indicates that KLF2 level both in gene expression and protein was up‐regulated in vivo in BM, spleen and thymus tissues after injection with HDACi either MS275 or TSA.

### 3.3 Effect of HDAC inhibitor on osteoclastic differentiation of monocytes

**Figure 3:**
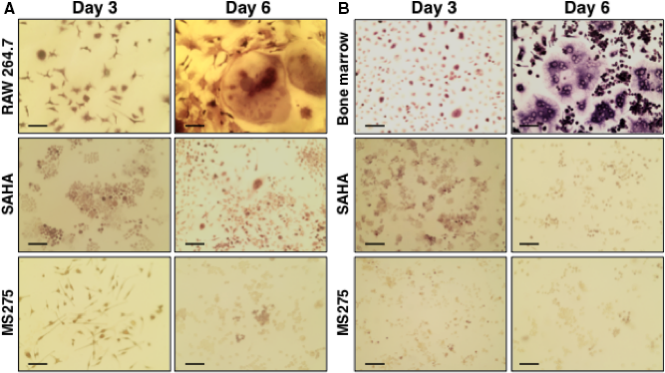
HDAC inhibitors reduce osteoclastic differentiation of monocytes. Murine monocytic cell line (RAW 264.7) (A) and murine primary bone marrow cells (B) were subjected to osteoclastic differentiation in the presence or absence of HDACi, such as SAHA (10 nmol/L) and MS275 (10 nmol/L). TRAP staining was performed during the course of osteoclastic differentiation at days 3 and 6. Images were depicted here. Scale bar indicates 10 μmol/L

We next sought to find whether HDAC inhibitors have any effect on monocyte differentiation to osteoclasts, as they are the important cells in arthritic tissue damages.^26^, ^27^ A murine monocytic cell line (RAW 264.7) and murine primary BM cells were stimulated in vitro using IL‐4 and sRANKL^14^ in the presence or absence of non‐toxic doses of HDAC inhibitors; SAHA (10 nmol/L) or MS275 (10 nmol/L). TRAP staining showed that the osteoclast differentiation of monocytic cells (Figure 3A) or primary BM cells (Figure 3B) was remarkably reduced in the presence of HDAC inhibitors. Monocytic cells and BM cells did not adhere to the culture plate and remained alive during the course of differentiation.

### 3.4 Factors associated with HDAC inhibitor‐mediated osteoclastic differentiation

**Figure 4:**
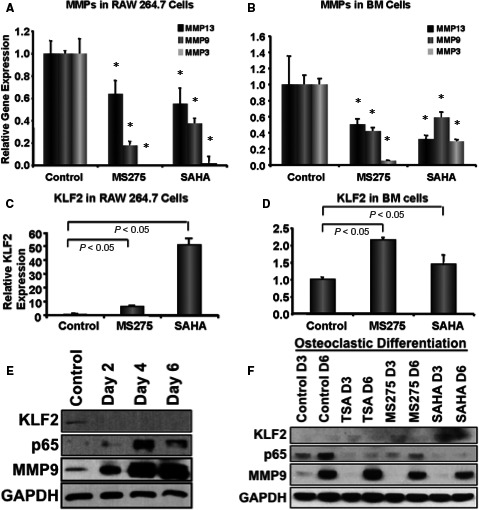
Reduced osteoclastic differentiation is associated with reduced levels of MMPs and increased level of KLF2. Total RNA was isolated from murine monocytic cell (RAW 264.7) (A, C) and murine primary bone marrow cells (B, D) that were subjected to osteoclastic differentiation in the presence or absence of HDACi, such as SAHA (10 nmol/L), MS275 (10 nmol/L) or TSA (10 nmol/L), and were subjected to real‐time RT‐PCR analysis for expression levels of MMP3, 9 and 13 along with KLF2 keeping GAPDH as an internal control, shown graphically. RT‐PCR experiments were performed in triplicate. The experiment was performed at least three times, and (*) indicates statistical significance (*P* < 0.05) when compared to the respective controls. (E) Western blot analysis was performed during the course of osteoclastic differentiation of RAW 264.7 cells for KLF2, NFκB (p65) and MMP9 proteins keeping GAPDH as an internal control. (F) Western blot analysis was performed during the course of osteoclastic differentiation of RAW 264.7 cells as indicated for KLF2, NFκB (p65) and MMP9 proteins keeping GAPDH as an internal control in the presence or absence of HDACi

To further investigate the reasons for HDAC inhibitor‐mediated reduced osteoclastic differentiation of monocytes, we analysed live cells in next sets of experiments. Real‐time PCR data revealed that the expression of MMP3, 9 and 13 were significantly reduced in both RAW 264.7 (Figure 4A) and primary BM cells (Figure 4B) in the presence of HDAC inhibitors either MS275 or SAHA compared to without HDAC inhibitors. Instead, KLF2 level was significantly higher in both RAW 264.7 (Figure 4C) and primary BM cells (Figure 4D) in the presence of HDAC inhibitors compared to controls. Similar results were observed in both days (days 3 and 6); data presented here is from day 6 of differentiation. Collectively, these data showed a direct correlation of up‐regulation of KLF2 associated with down‐regulation of MMPs in the monocytes in the presence of HDAC inhibitors. To confirm whether gene expression was translated to protein, Western blot analysis was performed; NFκB (p65) and MMP9 levels were increased during the course of osteoclastic differentiation (Figure 4E). However, when cells were treated with HDACi during the course of osteoclastic differentiation, MMP9 and NFκB (p65) levels were markedly decreased concomitantly KLF2 level was increased (Figure 4F). The Western blot data was quantified and shown graphically (Figure S2).

### 3.5 Effect of HDAC inhibitors in K/BxN serum‐induced arthritis in mice

**Figure 5:**
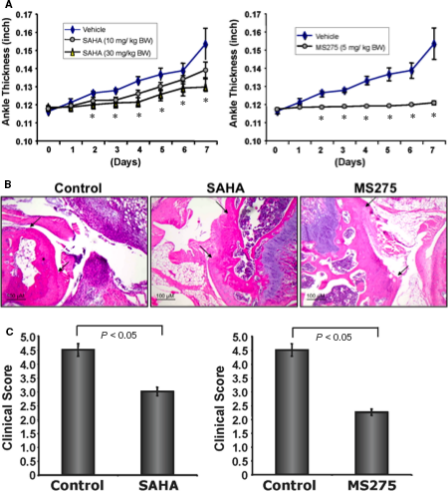
HDAC inhibitors reduce K/BxN serum‐induced arthritic inflammation, and bone and cartilage damage in mice. Group of C57BL/6 mice (8‐10 mice/group) were induced for development of arthritic inflammation using K/BxN serum (control), and were subjected to i.p. injection of HDACi (experimental; either SAHA, 10 mg/kg body weight; or SAHA 30 mg/kg body weight; or MS275, 5 mg/kg body weight) during induction of arthritis for 7 days. (A) Measured ankle inflammations are shown graphically during the development of arthritis for each inhibitor separately, and (*) indicate statistical significance (*P* < 0.05) when compared to the respective controls. (B) Haematoxylin and eosin staining of ankles (representative of four animals of each group) showed that reduced bone and cartilage damage after treatment with either of the HDAC inhibitors (SAHA or MS275). Arrowheads indicate the damaged area of the joints. (C) Clinical score of the histology showed that a damage of bone and cartilage were significantly reduced after treatment with SAHA or MS275 inhibitors

As HDAC inhibitor induced KLF2 in monocytes and reduced osteoclastic differentiation of monocytes, we next tested whether HDAC inhibitor has any effect on arthritic inflammation and severity in joint destruction in mice. Mice were induced arthritis using K/BxN serum and treated with HDAC inhibitors (either SAHA, 10 mg/kg body weight; or SAHA 30 mg/kg body weight; or MS275, 5 mg/kg body weight) during the course of arthritis development for 7 days. Ankles were measured, and found that a significant reduction in inflammation after injection of HDAC inhibitor with SAHA 30 mg/kg body weight; or MS275, 5 mg/kg body weight from day 2 to day 7 (Figure 5A). When the lower dose of SAHA (10 mg/kg body weight) was used as therapeutics, a marked reduction in ankle inflammation were also observed (Figure 5A, left panel). These data show that ankle inflammation could be reduced after HDAC inhibitor therapy to K/BxN serum‐induced mice.

### 3.6 Effects of HDAC inhibitors on bone and cartilage damage upon K/BxN serum‐induced arthritis

As HDAC inhibitor reduced ankle inflammation in K/BxN serum‐induced arthritic mice, we next sought to find whether bone and cartilage damage were also reduced. H&E staining revealed that a reduced bone and cartilage damage after treatment with either of the HDAC inhibitors like SAHA or MS275 (Figure 5B). The damage of bone and cartilage were significantly lower in animals (Figure 5C) that were treated with SAHA (30 mg/kg body weight) or MS275 (5 mg/kg body weight).

### 3.7 To determine interaction between KLF2 and HDAC

**Figure 6:**
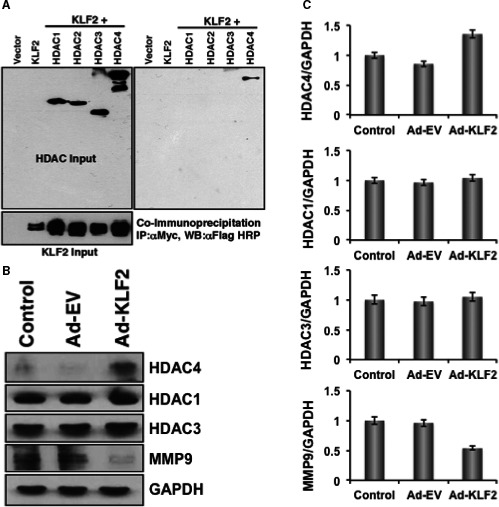
HDAC directly interacts with KLF2. (A) Co‐immunoprecipitation assays showed that KLF2 directly interacts with HDAC4 molecule. (B) Western blot data revealed that overexpression of KLF2 resulted in increased level of HDAC4 and concomitantly decreased level of MMP9, whereas, levels of HDAC1 and 3 were minimally changed. (C) Quantified Western blot data of stated molecules are presented graphically

To determine whether KLF2 and HDAC interact with each other in cells, we performed co‐immunoprecipitation assays by overexpressing KLF2 and HDAC1 through HDAC4 independently transfecting in COS‐7 cells. Results showed that KLF2 directly binds with HDAC4 in cells (Figure 6A). To confirm how KLF2 affects on HDAC molecules, KLF2 was overexpressed on RAW 264.7 cells and assessed the expressions of various HDAC molecules. The WB results showed that KLF2 overexpression resulted in higher level of HDAC4 and in decreased level of MMP9 (Figure 6B). There were very little changes in the levels of HDAC1 and 3. The Western blot data were quantified and shown graphically (Figure 6C). These results confirm that KLF2 directly interacts with HDAC molecule.

## 4 DISCUSSION

Rheumatoid arthritis is a progressive autoimmune disease with multifaceted pathobiology involving numerous cells, including monocytes, and various signalling pathways, to drive the inflammation.^28^ KLF2, a transcriptional factor, is important in regulating endothelial cell development, homeostasis and activation,^29^ and inhibiting proinflammatory activation of monocytes.^11^, ^14^ Therefore, delineating the regulatory role of KLF2 is very important in understanding the diverse roles of KLF2 in RA pathogenesis. On the other hand, HDACi‐mediated inhibition of inflammatory gene expression^30^ and immune cell activation^31^ was reported; however, the exact mechanism is yet to be defined. Therefore, we sought to investigate whether HDACi has any effect on KLF2‐mediated regulation of RA pathogenesis.

Among underlying processes in RA, KLF2‐mediated regulation of osteoclastic differentiation is critically important. Finding the molecules that regulate KLF2 in monocytes could be useful in managing RA pathogenesis. Herein, we found increased KLF2 expressions both in RNA and protein levels after induction with several HDACi molecules (Figure 1). We next tested HDACi effects on KLF2 expression in vivo. An increased level of KLF2 expression was found in murine BM, spleen and thymus tissues upon infusion with HDACi (Figure 2). Interestingly, in vitro differentiation of monocytes to osteoclasts is remarkably reduced upon HDACi addition (Figure 3). It is possible that the decreased capability of osteoclast differentiation was because of the up‐regulated KLF2 level in monocytes. Our finding shows that the up‐regulated KLF2 is not only decreased the proliferation of monocytes, but also reduced osteoclastic differentiation.

Out of many inflammatory signals, matrix‐proteolytic enzyme‐mediated signalling is highly responsible for destruction of cartilages, bones and articular structures.^32^ MMPs can cleave adhesion molecules, cytokines, chemokines, growth factors, and their receptors and binding proteins.^33^ Moreover, they also activate other proteases, and thus, mediate a cascade of severe matrix degradation process.^33^ Several MMPs are elevated in the synovial fluid and in the serum of RA patients.^34^ Herein, we show that reductions in osteoclastic differentiation upon HDACi addition to monocytes are associated with decreased expression of MMPs along with increased level of KLF2 (Figure 4). Therefore, as metalloproteinase proteolytic axis ensures appropriate initiation and stop signals during the inflammatory reaction. Importantly, we found a significant dose‐dependent reduction in ankle thickness upon HDACi with SAHA or MS275 administration to K/BxN serum‐induced arthritic mice (Figure 5). The damage of bone and cartilage was significantly reduced in HDACi‐treated arthritic mice compared to the untreated control arthritic mice. These data support HDACi's ability to exert anti‐inflammatory effect on K/BxN serum‐induced arthritic mice, which is in consistent with the earlier observations with different models.^35^ Taken together, these data not only show the efficacy of HDACi against RA but also suggests a potential for future therapeutic modality against RA.

Finally, to determine whether KLF2 and HDAC interact each other in monocytes and mediate an effect on inflammatory transcriptional regulation process. Our data suggests that there is a clear interaction between HDACi and KLF2 in the transcriptional regulation level. We assume that acetylation of histone 3 proteins might play a key role in the HDACi regulated transcription progression, as we found the H3 acetylation is induced by HDACi. Histone acetylation causes alteration in nucleosomal conformation,^36^ which can increase the accessibility of transcriptional regulatory proteins to chromatin templates.^37^ Our earlier published data suggests that histone acetylation might result in increased transcriptional activity in monocytes by facilitating p65‐ and c‐Fos/c‐Jun‐dependent transcriptional progression of NFκB and AP1 gene, even in the presence of KLF2.^11^, ^14^ However, transcriptional regulation did not lead to inflammatory protein synthesis, rather reduced. Because the level of KLF2 was increased in the presence of HDACi both in vitro as well as in vivo*,* that mediated inhibition to the inflammatory gene expression and protein synthesis. In addition, KLF2 directly binds to the HDAC4 in cells (Figure 6A) and overexpression of KLF2 in monocytes resulted in higher level of HDAC4, and mediated in decreased level of MMP9 molecule (Figure 6B). These data provide evidence that KLF2 and HDAC interacts each other in monocytes in the transcriptional level and protein level, and resulted in reduction of arthritic inflammation.

In sum, our data provide evidence that HDACi up‐regulate KLF2 in monocytes both in vitro and in vivo. In addition, HDACi significantly reduce osteoclastic differentiation along with up‐regulation of the KLF2 and concomitant down‐regulation of MMPs. Moreover, HDACi reduce K/BxN serum‐induced arthritic inflammation and joint destruction in mice in a dose‐dependent manner. Furthermore, KLF2 and HDAC4 interacts each other in monocytes. These findings provide first mechanistic evidence of HDAC‐HDACi/KLF2‐axis in reduction of arthritic inflammation.

## DISCLOSURE

The authors have no conflict of interest. The authors did not receive any fund from commercial sources.

## AUTHOR CONTRIBUTIONS

All authors were involved in drafting the article or revising it critically for important intellectual content, and all authors approved the final version to be published. Dr. Das and The Ohio State University, and Texas Tech University Health Sciences Center have full access to the data of the study and take responsibility for the integrity of the data and the accuracy of the data analysis. Study conception, design and manuscript writing: MD and HD. Acquisition of data: MD, DL, MJ, RA, SK and HI. Reagents, analysis and interpretation of data: HD, MD, DL, SK, RA, HI, VP and MKJ.
