# Supplementary figures and images for: Induction of Krüppel‐like factor 2 reduces K/BxN serum‐induced arthritis
Source: J Cell Mol Med. 2018 Dec 3;23(2):1386–95. doi: 10.1111/jcmm.14041 (PMC6349180; doi:10.1111/jcmm.14041)

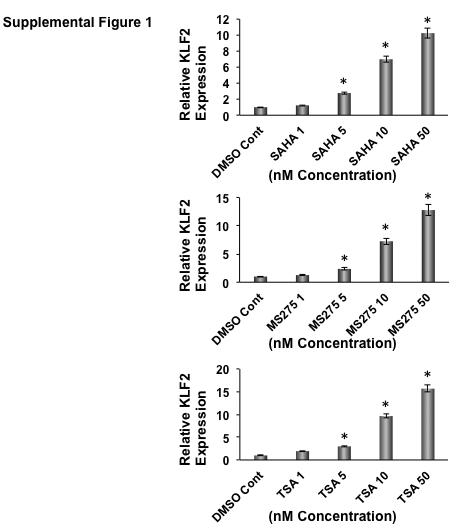

Supplement: Supplementary file 1 [file JCMM-23-1386-s001.tiff]

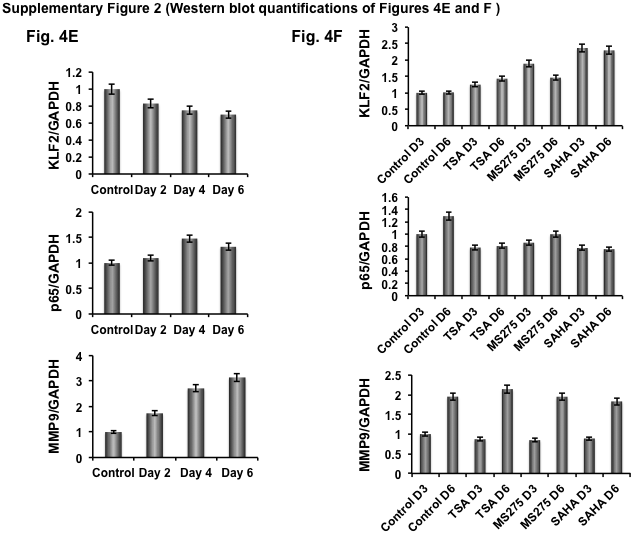

Supplement: Supplementary file 2 [file JCMM-23-1386-s002.tiff]
